# Reinforcement of Aminopropyl-Terminated Siloxane-Treated Carbon Nanotubes in Epoxy Thermosets: Mechanical and Thermal Properties

**DOI:** 10.3390/polym15153184

**Published:** 2023-07-27

**Authors:** Yuxin Sun, Xiwen Zhang, Dongyu Zhao

**Affiliations:** 1 School of Chemistry and Materials Science, Heilongjiang University, Harbin 150080, China; sunyuxin_xl@163.com (Y.S.); zxwrun@163.com (X.Z.); 2 Key Laboratory of Chemical Engineering Process & Technology for High-Efficiency Conversion, College of Heilongjiang Province, Harbin 150080, China

**Keywords:** amine functionalization, carbon nanotubes, nanocomposites, mechanical properties

## Abstract

The synthesis and characterization of aminopropyl-terminated polydimethylsiloxane- treated carbon nanotube (AFCNT)-reinforced epoxy nanocomposites are reported in the current study. The amine functionalization of the CNTs was performed with a reaction to PDMS-NH_2_. The AFCNTs were homogeneously dispersed in epoxy resin by using an emulsifier and a three-roller mill. The AFCNTs were characterized using Fourier-transform infrared spectroscopy (FTIR) and scanning electron microscopy (SEM). The curing behavior of the epoxy/AFCNT was studied using a differential scanning calorimeter (DSC). The tensile and impact strengths of the 2.0 wt.% AFCNT-reinforced epoxy nanocomposite were enhanced by 43.2% and 370%, respectively. Moreover, the glass transition temperature (*T_g_*) was also enhanced by 21 °C. Furthermore, significant enhancements were observed in the initial degradation and char yield values. SEM results confirmed that the AFCNTs were highly dispersed in the polymeric matrix.

## 1. Introduction

The invention and application of epoxy resins have brought great convenience to people’s lives [1]. Epoxy resins are widely used in industry for various applications, such as adhesives [2,3,4], coatings [5,6], packaging [7,8], and other structural materials [9,10,11]. The epoxy resins have a higher degree of crosslinks, thus showing thermosetting properties; for instance, higher degrees of rigidness and better strength and dimensional stability. However, the shortcomings of epoxy resin, such as its poor heat resistance and brittleness, has gradually begun to have an impact on its range of applications in the industry [12].

The carbon nanotube (CNT), a one-dimensional linear allotrope of carbon having a higher surface area, has attracted exceptional attention since its discovery, due to its outstanding mechanical and thermal properties [13,14]. Numerous studies have been done to improve its mechanical and thermal properties by reinforcing this promising nanomaterial in the field of polymer composites [15,16,17]. However, the blending of the CNT in the polymeric matrix is quite difficult due to the existence of its own strong van der Waals force and the formation of a very serious self-agglomeration. Various physical dispersion and chemical modification methods have been used to prevent the agglomeration of CNTs [18,19]. These methods include functionalization by covalent methods, non-covalent methods, polymers, and biomolecules and decoration with nanoparticles and surfactants [20]. Researchers reported that the dispersion and synergy of CNTs can be improved within the matrix resin by surface functionalization [21]. It has been demonstrated that functionalized CNTs as effective reinforcing fillers offer the possibility to achieve a good balance between high thermal stability and excellent mechanical properties of epoxy composites [20,22]. The CNT’s surface was chemically modified with vitamin B2 under microwave irradiation and reinforced in biodegradable poly(ester-imide) by Mallakpour and Soltanian [23]. Similarly, in another study by Mallakpour and Zadehnazari, CNTs were acid-modified and provided reinforcement in poly(amide–imide) [24]. Moreover, CNTs were functionalized with poly-L-lysine by a non-covalent method with N-carboxy anhydride polymerization by Ling and coworkers [25]. Donchak and coworkers observed that NH_2_-modified CNTs can be easily dispersed in a thermoplastic matrix [26]. In addition to this, the mechanical properties of the produced nanocomposites with NH_2_-modified CNTs were much better than those with non-modified CNTs containing nanocomposites. Based on these works, improvement of the properties of CNT polymer matrix composites largely depends on the good compatibility between the CNT and polymer matrix. The better the compatibility, the higher the dispersion and the more obvious the macroscopic performance change. Therefore, it is crucial to solve the agglomeration problem of CNTs and improve their dispersion in the polymer matrix.

In the present work, we proposed a new method to deal with the process complexity and irreversible agglomeration caused by the presence of varying degrees of drying. The CNTs were functionalized by using aminopropyl-terminated polydimethylsiloxane. Later, the epoxy matrix and treated CNTs were uniformly mixed by using an organic solvent. The mixture was dried and milled to achieve a uniform dispersion of the CNTs in the matrix. The mechanical properties and heat resistance of the nanocomposites were systematically studied.

## 2. Experiment

### 2.1. Materials

**Scheme 1:**
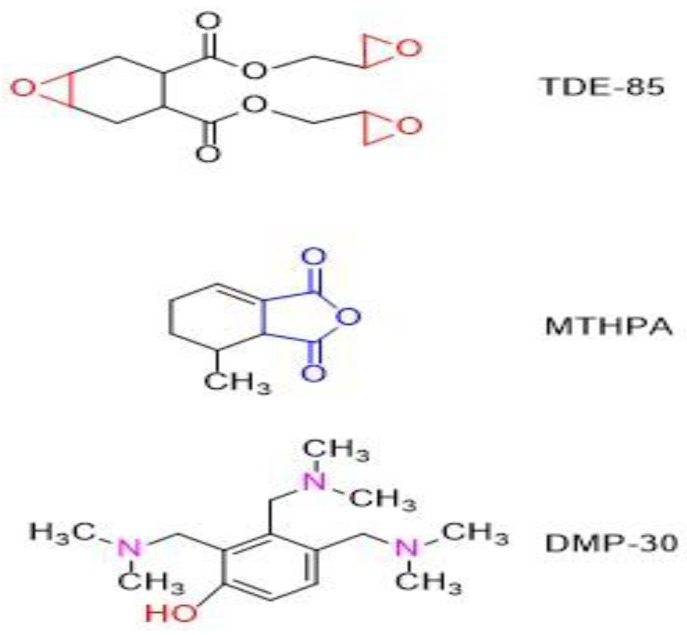
Chemical structures of resin, hardener, and accelerator used in the study.

A 4,5-epoxycyclohexane-1,2-dicarboxylic acid diglycidyl ester (TDE-85) epoxy matrix was supplied by Tianjin Jingdong Chemical Composites Co., Ltd. (Tianjin, China). Methyltetrahydrophthalicanhydride (MTHPA) and 2,4,6-tris(dimethylaminomethyl) phenol (DMP-30) were purchased from Zhejiang Alpha Chemical Technology Co., Ltd. (Zhejiang, China). CNT with a 20–40 nm diameter and about 5–15 μm length was purchased from Shenzhen Nanotech Port Co., Ltd., (Shenzhen, China). Aminopropyl-terminated polydimethylsiloxane (PDMS-NH_2_) was purchased from Zhengzhou Alfa Chemical Co., Ltd. (Zhengzhou, China). Sulphuric acid (H_2_SO_4_) and nitric acid (HNO_3_) were supplied by Tianjin Kemiou Chemical Reagent Co., Ltd. (Tianjin, China). N,N’-dicyclohexylcarbodiimide (DCC) was supplied by Shanghai Aladdin Chemical Reagent Co., Ltd. (Shanghai, China). Ethanol, acetone, and N,N’-dimethylformanide (DMF) were purchased from Tianjin City Fu Yu Fine Chemical Co., Ltd. (Tianjin, China). The chemical structures of the resin, hardener, and accelerator are shown in Scheme 1.

### 2.2. Synthesis of CNT-COOH

CNTs were treated with an H_2_SO_4_ and HNO_3_ mixed acid solution with a volume ratio of 3:1. The reactants were mechanically stirred for 3 h at 70 °C to implant the oxide groups on the CNT surface. The suspension was cooled to room temperature, and washed several times with distilled water until the filtrates’ pH value became neutral. The filter cake was transferred to a freeze dryer and dried for 12 h to obtain oxidized carbon nanotubes (CNT-COOH).

### 2.3. Preparation of Amino-Functionalized CNT

**Scheme 2:**
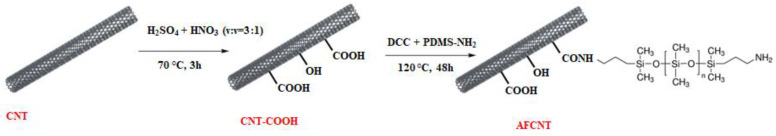
Surface activation mechanism of CNT.

CNT-COOH 0.05 g, PDMS-NH_2_ 10 mL, DMF 15 mL, and DCC 0.8 g were added into a 250 mL round bottom flask. The refluxing reaction was carried out at 120 °C for 48 h. After that, the excess raw materials and other by-products were washed away by ethanol under ultrasonic oscillation. Amino-functionalized CNT (AFCNT) was prepared from the filter cake by freeze-drying for 8 h and dried for 12 h in a vacuum oven at 60 °C. Scheme 2 shows the various steps involved in the CNT surface activation.

### 2.4. Preparation of the AFCNT/Epoxy Nanocomposites

**Scheme 3:**
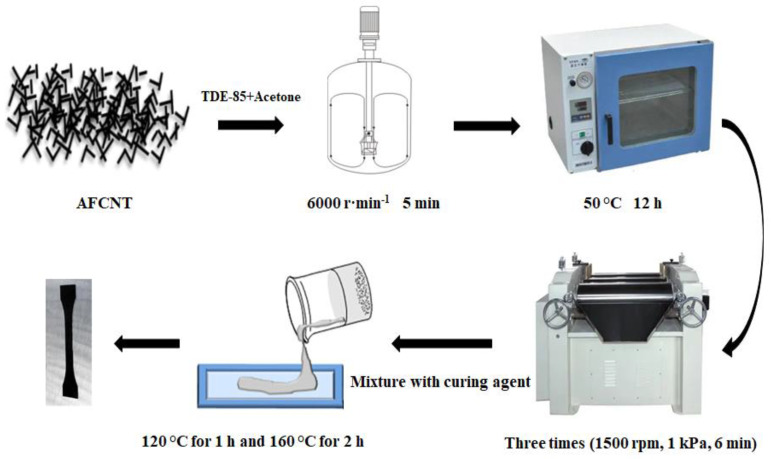
Schematic illustration for the preparation of AFCNT/epoxy nanocomposites.

AFCNT-reinforced epoxy nanocomposites were prepared by blending and grinding with solvent. Appropriate amounts of AFCNT nanofiller and epoxy resin (TDE-85) were weighed and blended using a high-shear emulsifying machine (Shanghai ELE Mechanical and Electrical Equipment Co., Ltd., Shanghai, China) at 6000 RPM for 5 min, and acetone was added as a solvent for better dispersion of the AFCNT in the resin. Later, the solvent was removed from the mixture in a vacuum oven at 50 °C for 12 h. The uniform dispersion of AFCNTs in the resins was confirmed by grinding the AFCNT/resin blend in a three-roller mill three times. The addition of the curing agent with a 1:1 weight ratio of resin to curing agent was made by a THINKY centrifugal mixer (ARV-310, Tokyo, Japan) vacuum mixer (1500 RPM, 1 kPa, 6 min). Finally, the mixture was poured into preheated steel molds and cured at 120 °C for 1 h and 160 °C for 2 h. The reinforcement of AFCNT nanofillers was varied from 0.5 to 2.5 wt.%, with a regular interval of 0.5 wt.%. During the AFCNT blending, the viscosity of the blend was continuously increased and reached the maximum level at 2.5 wt.% AFCNT blending. Therefore, a further increase during the reinforcing phase was not carried out. For the comparative analysis, the neat epoxy resin sample was cured with a similar procedure; however, AFCNT nanofiller was not added. Scheme 3 represents the various steps involved in the manufacturing of nanocomposites.

### 2.5. Characterization

Fourier-transform infrared (FTIR) spectra analyses were performed at room temperature in the range of 4000–400 cm^−1^ by using the transmission mode (BRUKER TENSOR-27).

The fracture surface morphologies of the pristine matrix and epoxy nanocomposites were examined by using scanning electron microscopy (SEM) (JSM-IT300, JEOL, Peabody, MA, USA. The fracture surface samples were placed in liquid nitrogen and sputter-coated with a thin layer of gold to reduce any charge build-up on the surface.

Differential scanning calorimetric (DSC) analysis was recorded on a Q20 of TA Instrument (New Castle, DE, USA) from 20 to 200 °C under an N_2_ atmosphere at a heating rate of 20 °C·min^−1^.

The tensile strength tests of the pristine epoxy matrix and nanocomposites were measured on an Instron 5982 electronic tensile machine at room temperature and a rate of 2 mm·min^−1^. Standard dumbbell-shaped specimens (100.0 mm × 12.5 mm × 4.0 mm) were made by following the ASTM D-638 standard for the tensile test. The impact strength of an 80 mm × 10 mm × 4 mm sample size was tested on a PTM 1000 material tester of Shenzhen Sansi Technology Co., Ltd., (Shenzhen, China). For all the mechanical tests, at least five specimens for each single composition were tested.

Thermal stabilities from 50 to 750 °C under an air atmosphere of the neat epoxy matrix and nanocomposites were evaluated on a Q500 Thermo gravimetric analyzer from TA instruments (New Castle, DE, USA) at a 10 °C·min^−1^ heating rate.

The glass transition temperature (*T_g_*) was estimated from the tan delta peak temperature by studying the thermomechanical properties on a Dynamic Mechanical Analyzer (DMA) Q800, TA instruments (New Castle, DE, USA). Tests were performed in temperatures ranging from room temperature to 250 °C in single cantilever mode at 1 Hz with a constant heating rate of 5 °C·min^−1^.

## 3. Results and Discussion

### 3.1. Characterizations of CNTs

**Figure 1:**
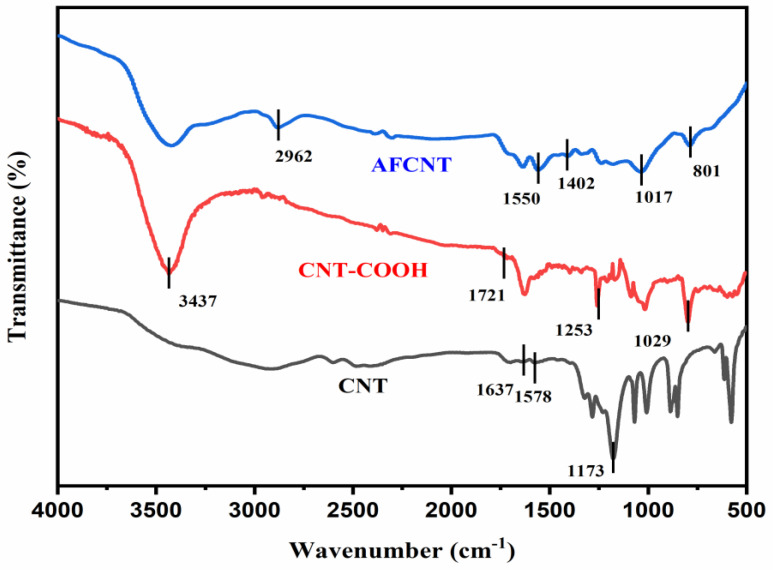
FTIR spectra of pristine, intermediate, and amino-functionalized CNTs.

In order to verify the presence of PDMS groups on the top surfaces of functionalized CNTs, a series of FTIR tests were conducted, and the results are shown in Figure 1. In the figure, the three curves represent the CNT, CNT-COOH, and AFCNT samples.

The CNT sample curve showed three absorption peaks at 1637, 1578, and 1173 cm^−1^, respectively. Among them, the absorption peaks at 1637 and 1578 cm^−1^ were C=C stretching vibration peaks, and the 1173 cm^−1^ was the stretching vibration absorption peak of C-C, which can be attributed to the C-C stretching vibration of the internal carbon nanotubes.

In the CNT-COOH sample curve, the absorption peaks at 3437 and 1721 cm^−1^ were the stretching vibration peaks of -OH and C=O, respectively [27]. The absorption peaks at 1253 and 1029 cm^−1^ can be assigned to the C-O telescopic vibration in the O-CNTs [28,29]. These indicate that oxygen-containing functional groups such as hydroxyl, carboxyl, and others are formed on the CNT surface by oxidation treatment [27].

The AFCNT curve absorption peaks recorded at 2962 and 1017 cm^−1^ can be easily related to the C-H bonds and Si-O-Si bridge, respectively. The weak symmetric peak at 1402 cm^−1^ is related to CH_3_ [30]. Also, the characteristic peak at 3437 cm^−1^ was weakened, due to the formation of amide bonds between CNT-COOH and PDMS-NH_2_. The absorption peaks at 1550 and 1402 cm^−1^ can be assigned to the -NH- bending vibration and the C-N telescopic vibration, respectively. These observations indicate that the CNT-COOH are enriched with oxygen-containing groups after treatment, which facilitates the chemical interactions between CNT-COOH and PDMS-NH_2_.

**Figure 2:**
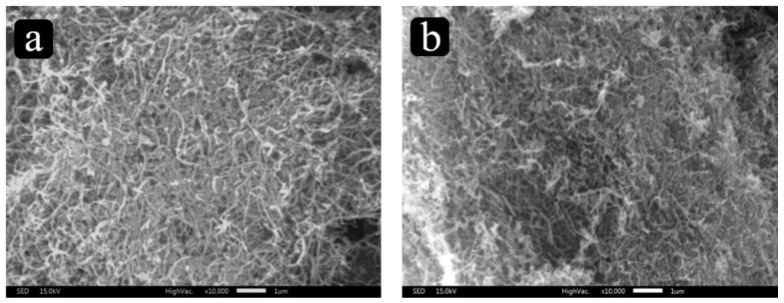
SEM micrographs of (**a**) CNT-COOH and (**b**) AFCNT.

Substances with a large surface area, such as CNT or graphene, tend to aggregate by themselves, due to the strong van der Waals forces and Coulomb attractions, to form an inevitable lamination, especially during the curing of polymeric resin. SEM images are the most direct means of representing the dispersion of the material. The morphology and microstructure of acidified CNTs (CNT-COOH) and AFCNT were confirmed by SEM, and the obtained morphological images are illustrated in Figure 2.

In Figure 2a, we can easily observe that the acidified CNTs have good dispersion, but also a small degree of inter-tube entanglement, and are ready for the next step, the amino modification of the CNTs. From Figure 2b, it can be seen that the outer layers of AFCNTs after PDMS-NH_2_ modification were partially coated with the modified layer, which makes the CNT coarser. This is because the previous acidifying treatment of the CNT made it more end-defective and very easily chemically modified, resulting in PDMS-NH_2_ grafting onto CNT.

### 3.2. Curing Behavior of AFCNT/Epoxy Blends

**Figure 3:**
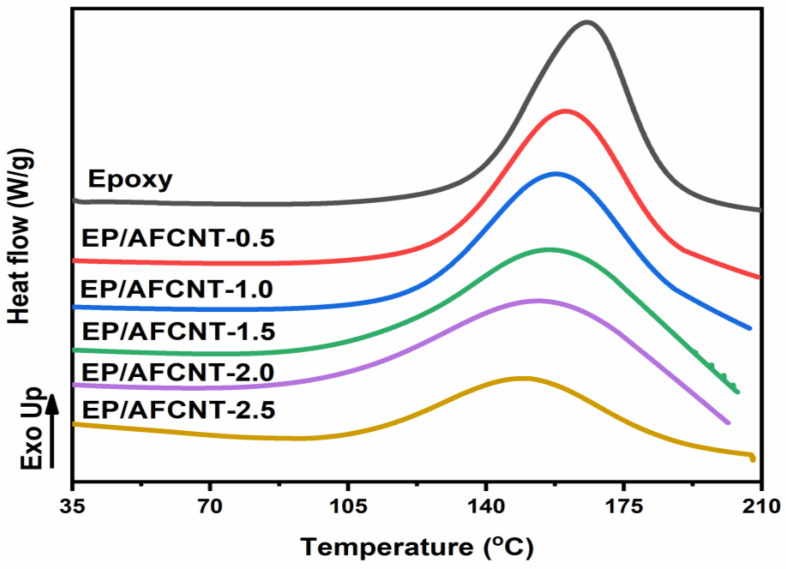
DSC spectra of pristine epoxy thermosets and AFCNT/epoxy blends having various AFCNT wt.%.

The curing behavior of AFCNT/epoxy blends with different wt.% of AFCNT blending was studied by DSC. The produced DSC curves and data from the AFCNT/epoxy blend are presented in Figure 3 and Table 1, respectively.

The produced non-isothermal DSC spectra of pristine epoxy thermosets and AFCNT/epoxy blends having various AFCNT wt.% indicated that the curing peak (**T_p_**) gradually weakened and moved towards lower temperatures by increasing the AFCNT blending. This drop in temperature confirms the catalytic effect of –NH_2_ groups being attached to AFCNTs [31]. Moreover, the onset curing temperature (**T_i_**) was also reduced from 125 to 98 °C as the AFCNT blending was raised. In addition, the curing reaction enthalpy (∆H) was gradually decreased from 216.4 to 180.6 J/g as the AFCNT blending was increased from 0 to 2.5 wt.%, respectively. Initially, the decline in ∆H was higher, then became slow for >1.0 wt.% blending. This indicates that the higher viscosity of the blends reduces the movement of chain segments and curing reaction [32]. This can be related to the higher energy demand of the epoxy system with the increase in AFCNT blending for the completion of the curing reaction.

### 3.3. Mechanical Properties

**Figure 4:**
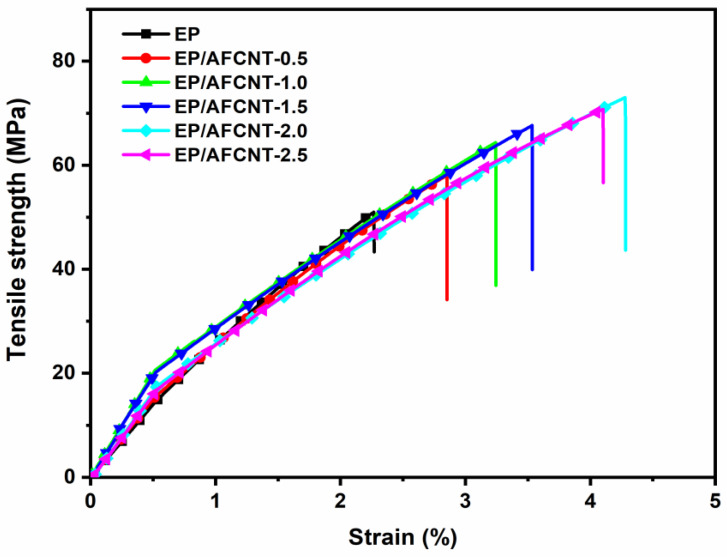
Tensile strength–stress curves of the pure epoxy resin and AFCNT-reinforced epoxy nanocomposites.

The tensile properties and impact intensity of the pure epoxy resin and AFCNT-reinforced epoxy nanocomposites on several AFCNT wt.% reinforcements were studied. Produced tensile strength plots are depicted in Figure 4 and the data extracted from the tensile plots and impact strength values are produced in Table 2.

The results showed that with an increase in the AFCNT reinforcement, the tensile strength and modulus values of the nanocomposites first increased and then decreased after attending the optimized loading. A 43.2% improved tensile strength as compared to the recorded value for the pristine matrix was observed with a 2 wt.% AFCNT reinforcement. Tensile strength recorded values for the pristine matrix and optimized-loading (2 wt.%) AFCNT-reinforced epoxy matrix were read as 50.98 ± 3.18 and 73.00 ± 1.72 MPa, respectively. Conversely, loading higher than the optimized amount reduced the tensile strength of nanocomposites to 70.75 ± 2.24 MPa. Meanwhile, with the increase in AFCNTs, the tensile moduli and strain values of the nanocomposites also showed similar behavior.

A similar enhancement in tensile strength was recorded by Mallakpour and Zadehnazari for acid-modified CNT-reinforced poly(amide-imide) that was cured after blending into the matrix by an ultra-sonication-assisted solution process [24]. The tensile strength of carboxyl-functionalized CNT-reinforced composites was enhanced by 47% upon a 15 wt.% carboxyl-functionalized CNT incorporation. Rahmanian and coworkers reinforced CNT-grown silica in an epoxy thermoset [33]. They reported a 1 wt.% of CNT–silica reinforcement as providing optimized loading, resulting in a 35% higher tensile strength value; moreover, further reinforcement of the CNT–silica showed a decline in enhancement.

The impact intensity of pure epoxy resin and AFCNT-reinforced nanocomposites are also summarized in Table 2. From the data results, we can easily observe that the impact resistance of the AFCNT-reinforced nanocomposites was greatly improved. The impact strength of the nanocomposites was nearly doubled by reinforcing just 0.5 wt.% of AFCNTs in the nanocomposites. After a 1.5 wt.% AFCNT reinforcement, the increase was very slow, as the dispersion was difficult in the resin. However, even a 2.5 wt.% of AFCNT-loaded nanocomposites showed an enhancing trend in the impact strength values. The recorded value (13.43 kJ/m^2^) was 370% higher than the pure epoxy resin value (2.86 ± 0.37 kJ/m^2^). These enhancements can be attributed to the uniform dispersion of CNTs in the resins, as the reinforced CNTs have better mechanical properties than the pristine matrix [34].

### 3.4. Thermal Properties

**Figure 5:**
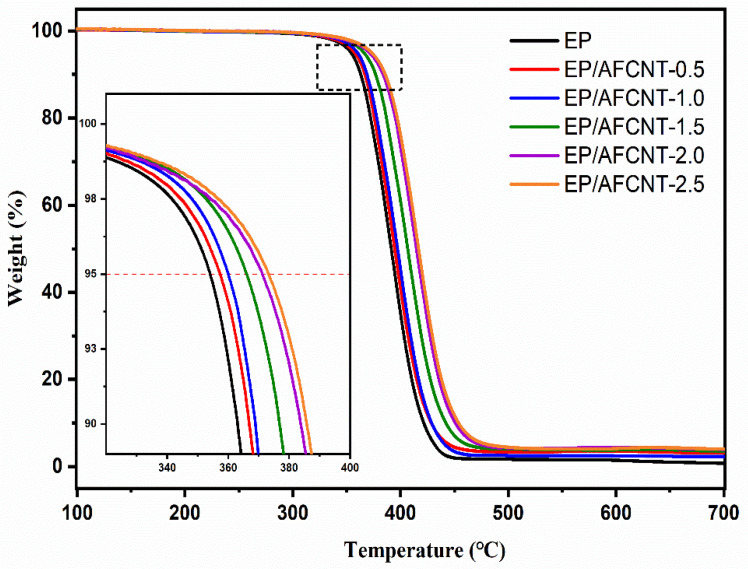
TGA curve of neat epoxy and its AFCNT-reinforced nanocomposites.

The thermal stability of epoxy and AFCNT-reinforced nanocomposites of various AFCNT wt.% in the air atmosphere was studied. Produced spectra are reported in Figure 5 and extracted data from the curves are summarized in Table 3.

Epoxy resin has excellent temperature resistance, and is commonly used in packaging materials and high-temperature adhesives [35]. The initial degradation temperatures are those that result 5 and 10 percent weight losses (T_5_ and T_10_); for the pristine matrix, these were recorded as 352 and 363 °C. After the reinforcement of the AFCNT nanoparticles in the epoxy matrix, the produced polymeric composites showed improved thermal stabilities; T_5_, T_10_, and char yield at 700 °C (Y_c_) values were gradually enhanced, and the best values were observed with 2.5 wt.% AFCNT nanoparticle loadings. The recorded values were 22 and 24 °C higher for T_5_ and T_10_, respectively. Similarly, the Y_c_ value was 3.35% higher than the corresponding value for the neat matrix. These enhancements confirm an improved dispersion and interface interaction of the AFCNTs within the matrix. The AFCNT nanocomposites could improve the barrier effect of CNTs and prevent the volatilization of degradation components [16]. This phenomenon has proven the effect of functionalization on the heat resistance of epoxy resin.

### 3.5. The Glass Transition Temperatures of Nanocomposites

**Figure 6:**
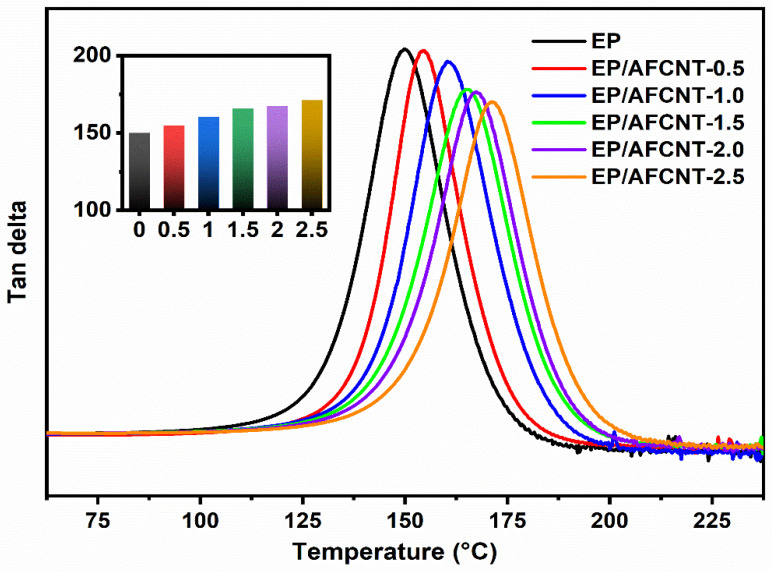
Temperature dependence of loss factor (tan delta) and glass transition temperature for epoxy resin and AFCNT nanocomposites.

The addition of filler can easily affect the glass transition temperatures (*T_g_*) of the nanocomposites [36]. The *T_g_* value can be determined from the *tanδ* curve produced during DMA analysis. Figure 6 presents the loss factor curve of the pure epoxy resin and AFCNT-reinforced nanocomposites on different AFCNT wt.%.

As can be observed, with a very low loading of AFCNTs, the *T_g_* value was significantly increased. The *T_g_* values for pristine matrix (unfilled) and 2.5 wt.% AFCNT-loaded nanocomposites were recorded as 149.9 and 171.2 °C, respectively. This suggests a cumulative rise of 14.21% in the *T_g_* value compared to the recorded value for the pure epoxy resin. Moreover, from the *T_g_* variation pattern produced in the histogram, we can summarize that the amino-functionalized CNT composites can effectively improve heat resistance.

This remarkable enhancement in the *T_g_* value as compared to the pristine epoxy matrix can be attributed to the AFCNT nanofiller having higher rigidity. Moreover, the tan delta peak height gradually reduced as the loading of the AFCNT nanofiller was raised from 0 to 2.5 wt.% due to the dilution phenomena. In addition to this, the relaxation process was also confirmed for the AFCNT-reinforced epoxy nanocomposites by the broadening of the tan delta peak.

The introduction of an AFCNT fraction in the nanocomposites restricted the molecular chain movement and relatively reduced free volume in the nanocomposites. Specifically, the good compatibility of the AFCNTs with the epoxy results in uniform dispersion of the nanoparticles, which may reduce the mobility of the polymer molecular chains [21]. The interfacial interaction between AFCNTs and the epoxy resin was further enhanced by the ability of the highly reactive group (-NH_2_) attached to the surface of AFCNT nanocomposites to participate in the epoxy resin curing reaction [37].

### 3.6. Interfacial Interaction Analysis by SEM

**Figure 7:**
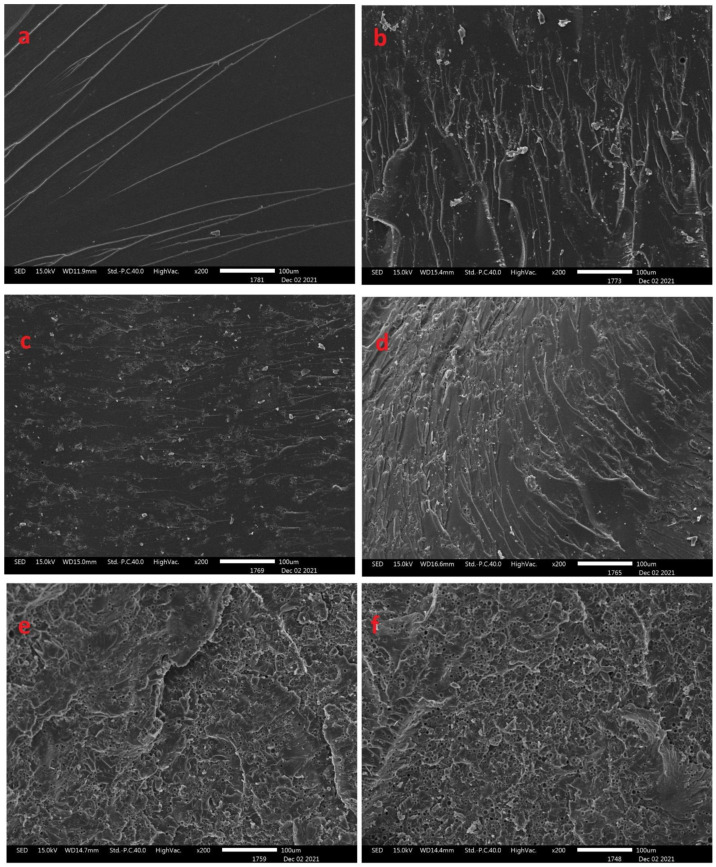
SEM micrographs of the fractured surfaces of resins: (**a**) neat epoxy, (**b**) 0.5, (**c**) 1.0, (**d**) 1.5, (**e**) 2.0, (**f**) 2.5 wt.% AFCNT-reinforced epoxy nanocomposites.

The SEM technique is one of the effective ways to evaluate the dispersion of nanofillers in the matrix and interface of nanocomposites. The fracture surface morphology of the nanocomposites observed using SEM is produced in Figure 7.

The pristine epoxy resin (Figure 7a) morphology shows linear sparse and parallel strip-like river-lines in the direction of crack propagation, confirming the brittle fracture characteristics of the epoxy resins [38]. After the addition of the functionalized AFCNTs in the matrix, the cross-sectional morphology of the material greatly changed, showing some characteristics of ductile fracture. Up to a 1.0 wt.% AFCNT content, the density of linear fringes increased and became discontinuous with the increase in AFCNT content. The change in the cross-section pattern morphology reflects that the functionalized CNTs had an effect on the crack-tip propagation path during the fracture process of the composite specimen. Thus, the energy of crack propagation was lost, and the fracture toughness of the nanocomposites was improved by increasing the energy required to achieve a continuous expansion [39].

With a 1.5 wt.% or more of AFCNT reinforcement, the change of the cross-sectional pattern was more obvious (Figure 7d–f); it was no longer parallel and straight, but was replaced by a lot of dimples, which were similar to fish scales. Moreover, a lot of stress whitening appeared at the edges of the dimples, showing better toughness. The reason for this is that PDMS molecules can be grafted onto the surfaces of the functionalized CNTs, which are compatible with the epoxy, and can also take part in the curing reaction of the matrix, thus producing good adhesion with the epoxy matrix. The energy required to destroy the interface is greatly increased, and the morphology of the interface is very different [40]. In addition, no agglomeration of CNTs were observed in any cross sections, which again proved that the dispersion of CNTs in the matrix was good. The tube structures of the stripped or pulled-out CNTs were also not observed on the cross section, indicating that the functional CNTs were completely wrapped by the epoxy, and the interface force between the CNTs and exoxy was very strong.

## 4. Conclusions

In this work, the CNT surface was functionalized by aminopropyl-terminated polydimethylsiloxane. Functionalization was confirmed by using FTIR and SEM techniques.

The DSC results revealed that the functionalized CNTs can accelerate the curing reaction of epoxy due to the effects of –NH_2_ on the AFCNTs. The AFCNTs were reinforced in the epoxy resin to produce the nanocomposites, and those were systematically investigated. The prepared nanocomposites showed significant improvements in mechanical properties and temperature resistance due to the effectively improved compatibility of AFCNT with the epoxy resin. The AFCNT-reinforced nanocomposites showed gradual enhancements in the *T_g_* as the loading was raised from 0 to 2.5 wt.%. The TGA results demonstrated that the AFCNT reinforcement significantly improved the temperature resistance of the epoxy resin and increased its thermal stability. SEM proved that the modified AFCNTs were more easily dispersed, and there was a stronger interfacial interaction between the nanofiller and matrix. The excellent mechanical properties and thermal stability of the nanocomposites confirmed that the surface modification method can be used to develop high-performance polymer materials.

## Figures and Tables

**Table 1:** DSC parameters of neat epoxy and AFCNT-reinforced nanocomposites.

AFCNT (wt.%)	T_i_ (°C)	T_p_ (°C)	∆H (J/g)
0	125	165	216.4
0.5	117	160	200.8
1	112	157	187.5
1.5	108	155	184.7
2	103	153	181.7
2.5	98	150	180.6

**Table 2:** Tensile and impact properties of neat epoxy and AFCNT-reinforced nanocomposites.

AFCNT Content (wt.%)	Tensile Strength (MPa)	Tensile Modulus (GPa)	Elongation at Break (%)	Impact Strength (kJ·m^−2^)
0	50.98 ± 3.18	3.29 ± 0.16	2.27 ± 0.21	2.86 ± 0.37
0.5	58.05 ± 2.24	3.48 ± 0.11	2.85 ± 0.18	5.67 ± 0.48
1	64.35 ± 3.87	3.66 ± 0.19	3.24 ± 0.17	7.66 ± 0.73
1.5	67.64 ± 2.69	4.00 ± 0.13	3.53 ± 0.20	11.69 ± 0.38
2	73.00 ± 1.72	4.10 ± 0.17	4.28 ± 0.14	12.75 ± 0.44
2.5	70.75 ± 2.24	4.02 ± 0.11	4.10 ± 0.21	13.43 ± 0.57

**Table 3:** TGA parameters of neat epoxy and AFCNT-reinforced nanocomposites.

AFCNT (wt.%)	T_5_ (°C)	T_10_ (°C)	Y_c_ (%) at 700 °C
0	352	363	0.71
0.5	357	367	2.31
1	359	369	3.11
1.5	366	376	3.54
2	371	383	3.94
2.5	374	387	4.06

## Data Availability

Not applicable.
